# Mucosa-Associated *Oscillospira* sp. Is Related to Intestinal Stricture and Post-Operative Disease Course in Crohn’s Disease

**DOI:** 10.3390/microorganisms11030794

**Published:** 2023-03-20

**Authors:** Shukai Zhan, Caiguang Liu, Jixin Meng, Ren Mao, Tong Tu, Jianming Lin, Minhu Chen, Zhirong Zeng, Xiaojun Zhuang

**Affiliations:** 1 Department of Gastroenterology, The First Affiliated Hospital, Sun Yat-sen University, Guangzhou 510080, China; 2 Department of Radiology, The First Affiliated Hospital, Sun Yat-sen University, Guangzhou 510080, China

**Keywords:** Crohn’s disease, intestinal stricture, intestinal fibrosis, *Oscillospira*, post-operative recurrence

## Abstract

Intestinal stricture remains one of the most intractable complications in Crohn’s disease (CD), and the involved mechanisms are poorly understood. Accumulating evidence suggests that the gut microbiota contributes to the pathogenesis of intestinal fibrosis. In this study, we investigated specific mucosa-associated microbiota related to intestinal strictures and their role in predicting postoperative disease course. Twenty CD patients who had undergone operative treatments were enrolled and followed up. Intestinal mucosa and full-thickness sections from stenotic and non-stenotic sites were sterilely collected. DNA extraction and bacterial 16s rRNA gene sequencing were conducted. Radiological and histological evaluations were performed to assess fibrosis. Microbial alpha diversity was significantly decreased in stenotic sites (*p* = 0.009). At the genus level, *Lactobacillus*, *Oscillospira*, *Subdoligranulum*, *Hydrogenophaga*, *Clostridium* and *Allobaculum* were decreased in stenotic segments (*p* < 0.1). The difference in *Oscillospira* sp. (stenotic vs. non-stenotic) was negatively correlated with the erythrocyte sedimentation rate (correlation coefficient (CC) −0.432, *p* = 0.057) and white blood cell count (CC −0.392, *p* = 0.087) and positively correlated with serum free fatty acids (CC 0.575, *p* < 0.05). This difference was negatively associated with intestinal fibrosis evaluated by imagological and histological methods (CC −0.511 and −0.653, *p* < 0.05). Furthermore, CD patients with a higher abundance of *Oscillospira* sp. in the residual intestine might experience longer remission periods (*p* < 0.05). The mucosa-associated microbiota varied between stenotic and non-stenotic sites in CD. Most notably, *Oscillospira* sp. was negatively correlated with intestinal fibrosis and postoperative disease course. It could be a promising biomarker to predict post-operative disease recurrence and a microbial-based therapeutic target.

## 1. Introduction

Crohn’s Disease (CD), one of the main subtypes of inflammatory bowel disease (IBD), is a lifelong relapsing and remitting inflammatory disease with increasing incidence and prevalence around the world [1]. More than 80% of patients are diagnosed in their youth, usually with terminal ileal and colonic involvement. A series of gastrointestinal and extraintestinal symptoms can present in the disease course of CD, leading to poor quality of life. With the development of intestinal inflammation and disease progression, intestinal fibrosis occurs over time, which ultimately results in multiple complications (intestinal stenosis, obstruction, or perforation), potentially requiring intestinal resection. Despite several biological agents showing efficacy in intestinal inflammation alleviation, effective anti-fibrotic therapies for intestinal stricture have yet to be developed [2]. Approximately 30–50% of patients eventually undergo fibrosis-associated surgeries 10 or more years following CD diagnosis, and some patients suffer from postoperative recurrence within a short period of time [3]. Nowadays, innovative therapies for the prevention or reversal of intestinal strictures have become an urgent challenge in the field of CD management.

Although a variety of genetic, immunologic, and environmental factors are considered to be jointly involved in intestinal stricture formation in CD, the exact pathogenetic mechanisms of fibrosis and stricture formation required further study. The gut microbiota, as an important environmental factor, has attracted increasing attention in recent years [4]. In our previous review, we summarized the causative and preventive effects of certain gut microbiota and metabolic products on intestinal fibrosis via inflammation regulation, fibroblast activation or differentiation, extracellular matrix formation, and so on. However, these data mainly come from animal experiments; reliable evidence from patients with CD is still very sparse [5]. Furthermore, most investigators have used fecal samples, which might be affected by various factors and fail to reveal the precise gut microbiota characteristics of particular sites [6]. 

Surgical excision is the primary therapy for CD patients with intestinal stricture, and these patients can experience different disease courses after the operation. Some patients might suffer from postoperative recurrence within a short period of time [7]. Previous studies have reported that intestinal microbiota dysbiosis is associated with postoperative infections and disease recurrence [8,9]. However, the results have been inconsistent and microbial predictors for postoperative courses have rarely been explored. To facilitate the use of the gut microbiota in improving the diagnosis and treatment of postoperative CD, it is imperative to elucidate the bacteria that are associated with disease recurrence and to evaluate whether these microbial factors can predict postoperative CD recurrence. 

Among the thousands of millions of intestinal bacteria, *Oscillospira* sp., a Firmicute from the Ruminococcaceae family, has received increasing attention. It is a type of Gram-positive bacteria with a slow growth rate, as calculated by the number of transfer RNA genes. No subordinate species could be successfully cultured for a long time [10,11]. However, with the development of next-generation sequencing for bacterial 16S rRNA genes, samples from the gut and feces were tested and *Oscillospira* sp. was found to account for a high proportion of the intestinal microbiota. This indicated *Oscillospira* sp. might play an important role in maintaining microbial flora balance and human health [12]. *Oscillospira* sp. has been detected frequently in recent research, with involvements in leanness, gallstones, diabetes, and Parkinson’s disease [13,14,15,16]. Notably, its abundance showed a negative correlation with a series of inflammatory diseases, such as non-alcoholic steatohepatitis [17]. However, the relationships between *Oscillospira* sp. And inflammatory bowel diseases have only rarely been explored.

In this study, the main aim was to portray the mucosa-associated microbial characteristics of intestinal stricture in CD via a paired comparison between stenotic and non-stenotic sites. Furthermore, we aimed to identify the predictive value of recurrence-related microbiota in post-operative CD. The bacteria involved in intestinal stricture and postoperative disease recurrence might represent a promising precision treatment for CD patients undergoing ileocaecal resection.

## 2. Materials and Methods

### 2.1. Study Population and Data Collection

This study was conducted between March 2021 and September 2022 and approved by the institutional ethics committee of the First Affiliated Hospital of Sun Yat-sen University.

A total of 20 consecutive patients with CD who underwent intestinal resection were enrolled, and CD diagnosis was based on clinical history, symptoms, and/or colonoscopic findings. The inclusion criteria were as follows: (1) age ≥ 14 years; (2) ileal or ileocolonic involvement; (3) patients with intestinal stricture who received ileocolonic resections in the absence of dysplasia or cancer. The exclusion criteria were as follows: (1) had antibiotic treatment during the previous 3 months or >2 weeks after surgery; (2) any other disease or condition that might interfere with the study assessments (as judged by the investigator); (3) pregnant or lactating women. This study complied with the Declaration of Helsinki, and informed consent was obtained from all participants.

Clinical data of patients with CD were obtained from the electronic information system including demographic, clinical, laboratory, endoscopic, radiological and pathological features. All patients were followed up, and a colonoscopy was performed to assess the endoscopic recurrence according to the Rutgeerts score, about 6–12 months after surgery. According to previous research, patients were identified as experiencing postoperative disease recurrence if their CDAI score was ≥200 or there was an increase of ≥70 points from baseline (clinical recurrence), or their Rutgeerts score was ≥ i2 (endoscopic recurrence) [18].

### 2.2. Sample Collection

Mucosal samples were sterilely collected from the surgical specimens of the stenotic and non-stenotic sites (incisal edge). For each location sampled, one biopsy was collected and fixed in formalin for standard histopathology at the sampling institution for pathology diagnosis. Additional biopsies were taken for microbial sequencing and saved at –80 °C until processing [19].

### 2.3. Radiological Evaluation 

According to the approaches of previous studies, the normalized magnetization transfer (MT) ratio was applied to evaluate intestinal fibrosis by utilizing patients’ regular magnetic resonance imaging results. MT ratio pseudo-color maps were generated via an in-house Matlab script (Math Works; Natick, MA, USA) [20]. Different colors represented various extents of fibrosis in different kinds of tissues. The bowel segment with the most thickened intestinal wall was identified through evaluation by an experienced radiologist. After that, another radiologist who was blinded to the patients’ information drew regions of interest (ROIs) on the designated segment and muscles to measure the MT ratio. For a segment of full-thickness bowel wall, three ROIs of various sizes were drawn along the border of the intestinal wall. The MT ratio was calculated as follows: MT ratio (%) = [1 − (M_sat_/M_0_)] × 100. Therein, the M_sat_ and M_0_ are the signal intensities acquired with and without the off-resonance pre-pulse saturation, respectively [21]. Then, the average MT ratio of three ROIs in the bowel wall and muscles was recorded separately. Finally, the MT ratio of the affected bowel wall was divided by that of the muscle on the same image to calculate the normalized MT ratio [22].

### 2.4. Histological Evaluation

According to standard procedures, the formalin-fixed mucosal tissues were embedded in paraffin and sectioned, and then hematoxylin-eosin staining (HE) and Masson’s trichrome staining were carried out. All slides were independently reviewed by two expert pathologists at our institution. In addition, the fibrosis score and space volume expansion were assessed according to the reported criteria. The areas of fibroblast proliferation mixed with collagen and other matrix deposition, forming scar-like fibrous tissue, were separately evaluated in each layer under four views of ten times magnification (including the mucosa, submucosa, muscularis propria and subserosal adventitia) and scored from 0 to 3 according to the extent of fibrosis (none, <33%, 33~66%, >66%). The space volume expansion of each layer was evaluated and scored by comparing the stenotic intestinal segment to the paired non-stenotic segment (none, <2 times, 2~3 times, >3 times) [23].

### 2.5. DNA Extraction and 16s rRNA Gene Sequencing

Total genomic DNA samples were extracted from mucosal samples with an OMEGA Soil DNA Kit (OmegaBio-Tek, Norcross, GA, USA), following the manufacturer’s instructions. After that, the V3–V4 region of bacterial 16S rRNA genes was amplified via polymerase chain reaction (PCR). The forward primer was 338F (5′-ACTCCTACGGGAGGCAGCA-3′) and the reverse primer was 806R (5′-GGACTACHVGGGTWTCTAAT-3′). Sample-specific 7-bp barcodes were incorporated into the primers for multiplex sequencing. As a result, the PCR components contained 5 μL of buffer (5×), 0.25 μL of Fast pfu DNA Polymerase (5 U/μL), 2 μL (2.5 mM) of dNTPs, 1 μL (10 uM) of each forward and reverse primer, 1 μL of DNA Template, and 14.75 μL of ddH_2_O. The procedure and indexes of thermal cycling were as follows: initial denaturation at 98 °C for 5 min; followed by 25 cycles consisting of denaturation at 98 °C for 30 s, annealing at 53 °C for 30 s, and extension at 72 °C for 45 s; final extension of 5 min at 72 °C. PCR amplicons were purified with Vazyme VAHTSTM DNA Clean Beads (Vazyme, Nanjing, China) and quantified via at Quant-iT PicoGreen dsDNA Assay Kit (Invitrogen, Carlsbad, CA, USA), after which they were pooled and multiplexed at equal concentrations. Raw pair-end 2 × 250 bp sequencing was performed through the Illlumina NovaSeq platform (Illumina, San Diego, CA, USA) with the NovaSeq 6000 SP Reagent Kit at Personal Biotechnology Co., Ltd. (Shanghai, China) [9].

### 2.6. Bioinformatics and Statistical Analysis

Statistical analysis was performed with SPSS 20 (IBM, Armonk, NY, USA) and R version 4.1.0 (R Foundation for Statistical Computing, Vienna, Austria), and the microbiome bioinformatics were performed with QIIME2 [24]. In the matter of microbiome bioinformatics, raw sequence data were demultiplexed, and then quality filtered, denoised, and merged using the DADA2 plugin [25]. Non-singleton amplicon sequence variants (ASVs) were aligned with MAFFT and rarefied to ensure further analysis at the same level for every sample [26]. ASV-level alpha diversity metrics (Shannon, Simpson) were estimated [27,28]. Beta diversity analysis was also performed (Jaccard distance) and visualized via principal coordinate analysis (PCoA) [29]. Taxonomy was assigned to ASVs using the classify-sklearn naive Bayes taxonomy classifier against the SILVA Release 132 Database. Random forest was applied to discover the most distinguishing identifiable components between different groups at every taxonomic level [30].

Normally distributed continuous variables were presented as means ± SE, and the non-normally distributed variables were presented as medians and ranges. Categorical variables were presented as frequencies. Paired *t*-test was performed to explore the difference between samples from different sites in every patient for those variables that fit a normal distribution, such as the comparisons of Shannon or Simpson indexes. If variables were non-normally distributed, the paired Wilcoxon signed-rank test was used, such as when analyzing the relative abundance of bacteria. The Spearman rank correlation test was applied to analyze the degree of correlation between bacteria abundance and indexes of the severity of illness. Remission maintenance in the postoperative time period was estimated by the Kaplan-Meier method, and the results were visualized by Kaplan-Meier curves. Unless otherwise noted, two-tailed statistical significance was established if *p* ≤ 0.05.

## 3. Results

### 3.1. Patient Characteristics

Twenty consecutive patients with CD who underwent resection for intestinal stenosis were enrolled in this study. The average age was around 40 years old, and most of the patients (60%) were male. The medium disease course was 30 months (ranging from 3 to 204 months). The included patients received diverse therapies before surgery, including immunosuppressants (35%), biologics (40%), and more. Two CD patients (10%) were treatment-naive. All patients presented ileal stenosis and received ileocecal resection.

Mucosal biopsies were collected from the resected tissue at the time of surgery. Additionally, sections from adjacent tissue corresponding to the origin of the biopsies for microbial analyses were histologically defined as stenotic and non-stenotic by a clinical pathologist. The clinical characteristics are summarized in Table 1. 

### 3.2. Mucosa-Associated Microbiota in the Stenotic Sites Differed from the Non-Stenotic Sites 

**Figure 1:**
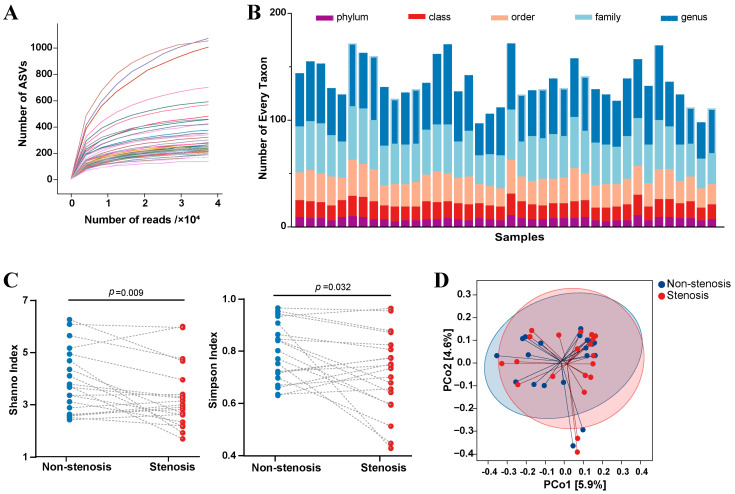
Mucosa-associated microbiota variations between intestinal stenotic and non-stenotic sites in CD. (**A**): Dilution curve representing the relationship between number of gene reads and amplicon sequence variants. Every curve represents a sample; (**B**): Number of different taxonomic levels in every sample; (**C**): Alpha diversity variations in different sites of every patient; (**D**): Principal Coordinates Analysis between samples from stenotic and non-stenotic sites.

A total of 20 paired intestinal mucous samples from stenotic and non-stenotic sites were collected for microbiome bioinformatics analysis. A medium sequencing read depth of 63,153 reads per sample (ranging from 39,459 to 79,835), including 8680 ASVs, was identified. Moreover, the dilution curve showed that all samples were sequenced with sufficient depth (Figure 1A). After rarefaction, 37,486 reads per sample and 8320 ASVs were randomly selected for further analysis. At the taxonomy level, 299 phyla, 602 classes, 971 orders, 1572 families, and 1986 genera were identified (Figure 1B). In terms of the microbial composition, the alpha diversity of mucosa-associated microbiota was decreased in the stenotic sites when compared to non-stenotic sites (Shannon index: 3.27 ± 1.23 vs. 3.90 ± 1.22, paired *t*-test *p* = 0.009; Simpson index: 0.72 ± 1.15 vs. 0.79 ± 0.11, paired *t*-test *p* = 0.032) (Figure 1C). However, beta diversity, assessed by Principal Co-ordinates Analysis, showed that mucosa-associated microbiota from these two groups were virtually indistinguishable (Figure 1D). Relative abundances of different bacteria in all mucosal samples were analyzed according to their different taxonomy levels, and the result was shown in Supplementary Materials Figure S1.

**Figure 2:**
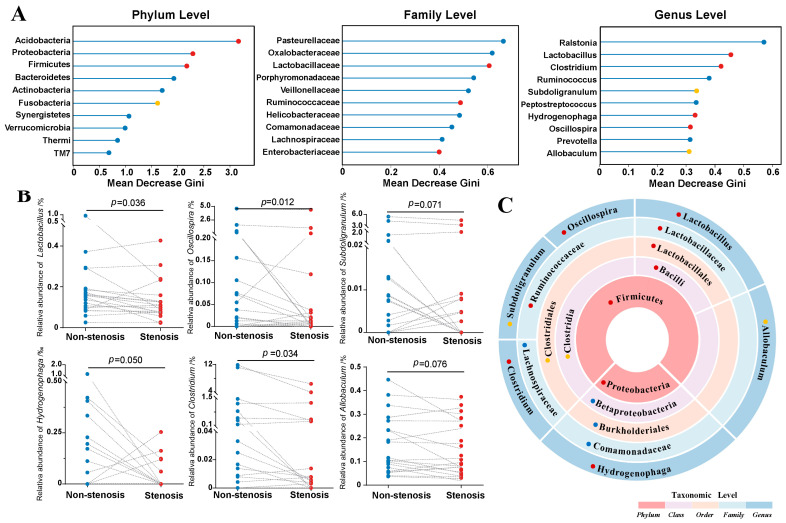
Taxonomic differences between intestinal stenotic and non-stenotic sites in CD. (**A**): Random forest analysis to identify distinguishing microbes at different taxonomic levels. The *x*-axis presented the contribution of each taxon to the microbial heterogeneity between stenotic and non-stenotic sites (shown by the mean decrease Gini index when the corresponding taxon was removed from the analysis model). (**B**): Bacterial abundance alterations in different sites of every patient. (**C**): Biological relationship of distinguishing taxonomic levels. Statistical results of relative abundance variation between two sites for the corresponding taxon: **● ***p* < 0.05; ● 0.05 < *p* < 0.1; ● *p* > 0.1.

In order to discover the bacteria with the greatest difference between the two groups, the random forest approach was applied to analyze distinctions at different taxonomic levels. *Acidobacteria*, *Proteobacteria*, *Firmicutes*, *Bacteroides*, *Actinobacteria*, *Fusobacteria*, *Synergistetes*, *Verrucomicrobia*, *Thermi*, and TM7 were the ten most distinguishing phyla between stenotic sites and non-stenotic sites. In addition, a significantly lower relative abundance of *Acidobacteria*, *Proteobacteria*, *Firmicutes*, and *Fusobacteria* was found in stenotic segments (*p* < 0.1). Similar analyses were also conducted at other taxonomic levels, and the results are shown in Figure 2A and Figure S2. At the genus level, *Lactobacillus*, *Oscillospira*, *Subdoligranulum*, *Hydrogenophaga*, *Clostridium* and *Allobaculum* were significantly decreased in the stenotic sites compared to the non-stenotic sites from the same patient (*p* < 0.1), indicating these microbes might be involved in the pathogenesis of CD and need further research (Figure 2B). Most notably, the taxonomic framework showed *Allobaculum*, *Clostridium*, *Lactobacillus*, *Oscillospira*, and *Subdoligranulum* belonged to the family (*Lachnospiraceae*, *Lactobacillaceae* and *Ruminococcaceae*), order (*Clostridiales* and *Lactobacillales*), class (*Clostridia* and *Bacilli*), and phylum (*Firmicutes*) levels with significantly different abundances as well (Figure 2C).

### 3.3. Mucosa-Associated Oscillospira sp. Was Negatively Associated with Intestinal Inflammation and Stenosis 

**Figure 3:**
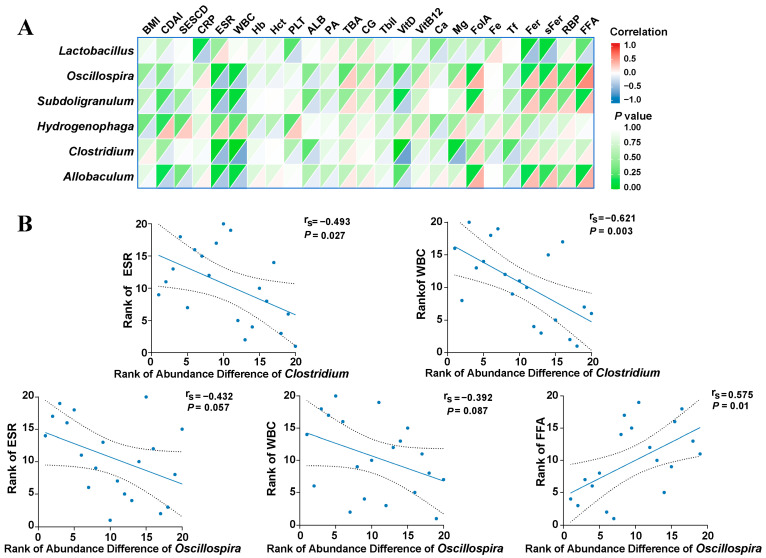
Correlations between bacterial abundance difference (stenotic site vs. non-stenotic site) and clinical indexes. (**A**): Heatmap showed the correlations between different genera and inflammatory indicators; (**B**): Spearman’s rank correlation analysis between Genus *Oscillospira* or *Clostridium* and clinical indexes. Every blue dot represents an enrolled patient and its location in the coordinate axis represents the rank of bacterial difference between two sites of this patients as well as the rank of his corresponding clinical index. BMI: body mass index; CDAI: Crohn’s disease activity index; SES-CD: simple endoscopic score for Crohn’s disease; CRP: C-reactive protein; ESR: erythrocyte sedimentation rate; WBC: white blood cell; Hb: hemoglobin; Hct: hematocrit; PLT: blood platelet; ALB: albumin; PA: prealbumin; TBA: total bile acid; CG: cholyglycine; Tbil: total bilirubin; VitD: vitamin D; VitB12: vitamin B12; FolA: folic acid; Tf: transferrin; Fer: ferritin; sFer: serum ferritin; RBP: retinol-binding protein; FFA: free fatty acids.

Considering that the relative abundance of *Lactobacillus*, *Oscillospira* sp., *Subdoligranulum*, *Hydrogenophaga*, *Clostridium*, and *Allobaculum* was lower in the intestinal stenotic sites, we hypothesized that these alterations might be related to disease activity and intestinal stricture to some extent. To test this hypothesis, the abundance variation was compared between the non-stenotic sites and the stenotic sites for every patient, and a correlation analysis was carried out between gut microbiota and inflammatory indicators (Figure 3A and Supplementary Materials Table S1). As shown in Figure 3B, the altered abundance of *Clostridium* (abundance in stenotic site—abundance in non-stenotic site) was negatively correlated with the erythrocyte sedimentation rate (ESR) (correlation coefficient −0.493, *p* < 0.05) and white blood cells (WBC) (correlation coefficient −0.621, *p* < 0.05). The same tendency could be found in the genus *Oscillospira* sp. (ESR: correlation coefficient −0.432, *p* = 0.057; WBC: correlation coefficient −0.392, *p* = 0.087), but a positive correlation was identified between *Oscillospira* sp. and the concentration of serum free fatty acids (FFA) (correlation coefficient 0.575, *p* < 0.05).

**Figure 4:**
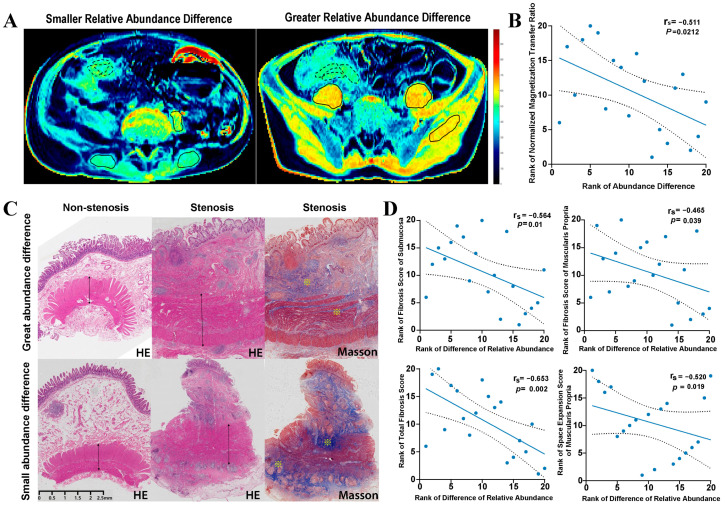
Correlations between Genus *Oscillospira* sp. abundance difference (stenotic site vs. non-stenotic site) and the degree of intestinal fibrosis. Patients with great and small *Oscillospira* sp. abundance differences were presented respectively. (**A**): Representative illustration showed the measurement of the normalized MT ratio of the affected bowel wall. Tissues with high MT ratios (such as skeletal muscle) appear yellow-red, while tissues with low MT ratios (such as subcutaneous fat) are dark blue. Normalized MT ratio of bowel wall was measured as MT ratios of the bowel wall (delineated by 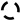)/MT ratios of muscle (delineated by 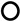); (**B**): Spearman’s rank correlation analysis between *Oscillospira* sp. abundance difference and normalized MT ratio of bowel wall; (**C**). Representative intestinal histopathological characteristics of stenotic and non-stenotic sites in the same patient. ↔ indicated space volume of muscularis propria layer; **※** indicated increased collagen deposition in submucosa and muscularis propria layers. (**D**): Spearman’s rank correlation analysis between *Oscillospira* sp. abundance difference and fibrosis score and space volume expansion score.

Furthermore, the correlation between gut microbiota alterations and the degree of intestinal fibrosis was also explored. Our research team verified that the normalized magnetization transfer (MT) ratio of the bowel wall, measured by magnetic resonance enterography, was a credible and noninvasive marker to estimate intestinal fibrosis in CD. Simply put, the normalized MT ratio is determined by the MT ratio of the bowel wall to the MT ratio of skeletal muscle in the same MR image (Figure 4A) [20]. We found that the abundance difference of genus *Oscillospira* sp. (abundance in a stenotic site minus the abundance in a non-stenotic site) was negatively associated with the normalized MT ratio of stenotic intestinal segments (correlation coefficient −0.511, *p* < 0.05) (Figure 4B and Supplementary Materials Table S1).

In addition, a similar correlation analysis was performed considering histopathological characteristics and the six distinguishing genera. For stenotic intestinal segments, the degree of fibrosis and space volume expansion were separately assessed in every layer (Figure 4C). As expected, the abundance difference of the genus *Oscillospira* sp. was negatively correlated with the total fibrosis score (correlation coefficient −0.653, *p* < 0.05). Regarding the specific intestinal layers, significantly negative correlations mainly occurred on the fibrosis scores of the submucosa (correlation coefficient −0.564, *p* < 0.05) and muscularis propria (correlation coefficient −0.465, *p* < 0.05). With respect to space volume expansion, we learned that the genus *Oscillospira* sp. was negatively associated with the expansion score of the muscularis propria layer (correlation coefficient −0.520, *p* < 0.05) (Figure 4D).

### 3.4. Mucosa-Associated Oscillospira sp. in the Residual Intestine Contributed to the Maintenance of Disease Remission

**Figure 5:**
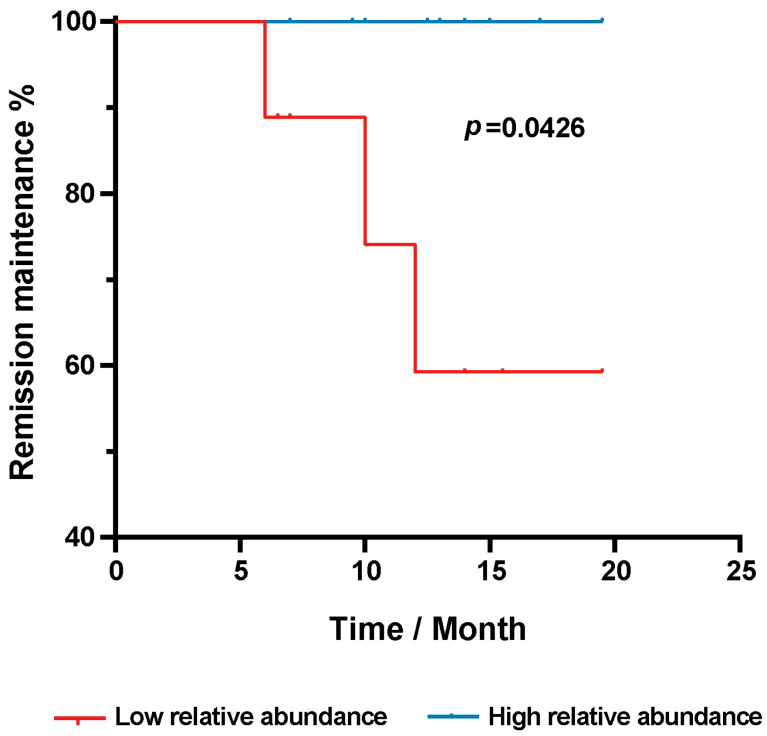
Kaplan-Meier’s curve showed the proportion of CD patients without disease recurrence over post-operative time. Comparison was conducted between patients with high and low abundance of mucosa-associated *Oscillospira* sp. in the residual segments.

Given that the genus *Oscillospira* sp. was identified as the most distinguishing microbe between the stenotic sites and non-stenotic sites, we considered the relationship between this genus and postoperative disease recurrence. We measured the relative abundance of the genus *Oscillospira* sp. in non-stenotic sites and in the residual intestine. According to the relative abundance of *Oscillospira* sp., patients with CD were equally divided into two groups: the low abundance group, containing nine patients with a relative abundance of *Oscillospira* sp. ranging from 0 to 0.021%; and the high abundance group, containing other eleven patients with a relative abundance ranging from 0.0381% to 4.6%. All patients were followed up to evaluate their postoperative course. Detailed information on bacterial abundance and follow-up data are summarized in Table 2. As shown in Figure 5, CD patients in the high abundance group were inclined to maintain disease remission for a longer period of time when compared to the low abundance group (*p* = 0.0426).

## 4. Discussion

Microbial characteristics of the intestinal mucosa of a cohort of CD patients undergoing fibrosis-associated surgeries were identified in this study, and we found that some specific microbes might be involved in intestinal stricture. Paired comparison between stenotic and non-stenotic sites in the same patient avoided confounding factors such as individual lifestyle, dietary patterns, and drug consumption. In addition, mucosal colonization microbiota allowed us to identify the specific bacteria involved in the pathophysiology of CD. We concluded that the bacterial diversity of mucosa-associated microbiota in the stenotic segment was decreased, especially the relative abundance of *Lactobacillus*, *Oscillospira*, *Subdoligranulum*, *Hydrogenophaga* and *Clostridium*. Moreover, we discovered the genus *Oscillospira* sp. was negatively correlated with the degree of intestinal stricture and associated with the maintenance of disease remission during the postoperative time period.

Although *Oscillospira* sp. has rarely been researched in CD, a meta-analysis reported its abundance was decreased in patients with CD, and it was negatively correlated with intestinal fibrogenesis in another animal study [31,32]. Here, we first searched on GMrepo, a curated database of human gut metagenomes (https://gmrepo.humangut.info/home (accessed on 1 October 2022)) and identified significantly decreased *Oscillospira* sp. in patients with CD when compared to the healthy controls (Supplementary Materials Figure S3) [33]. Moreover, we further ascertained that the reduction of *Oscillospira* sp. in the stenotic sites was more severe, indicating sustained *Oscillospira* sp. loss with the disease progress of CD. In addition, results from our study showed the decreased degree of *Oscillospira* sp. in stenotic sites was negatively correlated with the concentration of serum-free fatty acids, and positively correlated with intestinal fibrosis and some inflammatory biomarkers (ESR, WBC). To our knowledge, this was the first study to detect a negative correlation between *Oscillospira* sp. abundance and the degree of intestinal stricture/fibrosis in a CD patient cohort. The underlying mechanism of intestinal inflammation alleviation and fibrosis improvement might depend on the metabolite butyrate. The complete butyrate-kinase mediated pathway was identified in *Oscillospira* sp. via gene sequence information, indicating this germ might be a prospective butyrate producer and able to utilize gluconate originating from the host [34]. *Oscillospira* sp.’s butyrate production ability and the anti-inflammatory/epithelial protective role of butyrate have been comprehensively studied, and it has been confirmed that butyrate significantly prevents neutrophils from producing proinflammatory cytokines and chemokines [35,36]. It suppresses neutrophil migration and decreases the formation of neutrophil extracellular traps [37]. In terms of the fibrosis mechanism, butyrate was shown to suppress the extracellular matrix gene expression and decrease the abundance of alpha-smooth muscle actin and collagen in a model of human intestinal organoids [38]. More importantly, *Oscillospira* sp. was identified to be associated with the maintenance of post-operative remission in our study. Operation-treated patients with a decreased abundance of *Oscillospira* sp. in stenotic sites or residual intestinal segments had a higher risk of disease recurrence. CD is an autoimmune disease, and relapsing and remitting inflammation persists for the patient’s whole life. It is well known that persistent and overwhelming inflammation plays a vital role in post-operative recurrence [39]. As mentioned above, *Oscillospira* sp. has anti-inflammatory properties through its production of butyrate. Decreased abundance of *Oscillospira* sp. in residual intestinal segments might indicate more intense inflammation in the post-operative period and lead to relapsing. In that case, active follow-up and examination of these patients are recommended, to ensure that they benefit from treatment.

In this single-center study, the number of patients was small and we lacked validation of independent cohorts, but comparisons of paired samples helped to reduce selection bias. Consequently, further studies in *Oscillospira* sp. culturation are of great significance, and more detailed biological functions of *Oscillospira* sp. should be confirmed. Deeper verification of *Oscillospira* sp.’s effects on inflammation alleviation and intestinal stricture is required. In brief, the findings from this research suggest a novel microbial-based target for disease management and therapy in CD. Supplementation of this promising microbe in post-operative CD may be a strategy to prevent or treat disease recurrence.

## 5. Conclusions

The mucosa-associated microbiota in the stenotic sites was different from that in the non-stenotic sites. In addition, the distinguishing microorganisms were interrelated at different taxonomic levels. The genus *Oscillospira* sp. was negatively correlated with the degree of intestinal fibrosis and positively correlated with prolonged time of disease remission after surgery. Additionally, *Oscillospira* sp. also showed a positive correlation with the concentration of serum-free fatty acids and a negative association with serum inflammatory biomarkers. *Oscillospira* sp. regulates inflammatory and fibrosis processes as a butyrate producer. In general, our study indicated *Oscillospira* sp. could help to monitor the disease course and predict postoperative disease recurrence. Further studies on *Oscillospira* sp. culturation and biological functions are urgently needed, and its exact mechanisms in CD are of great significance.

## Figures and Tables

**Table 1:** Demographics and clinical characteristics of CD patients.

Cohort [*n*]	20
Age /years old	39.9 ± 13.43
Sex [male/female]	12/8
BMI /kg·m^−2^	16.80 ± 2.20
Smoker [*n* (%)]	3 (15%)
Disease duration/months [range; median]	3–204; 30
Regions of disease involvement [*n* (%)]	
Isolated ileal	5 (25%)
Ileocolonic	15 (75%)
Colonic	0 (0%)
Proximal small intestine or stomach or esophagus	8 (40%)
Perianal disease	10 (50%)
Stricture site	
Ileum only [*n* (%)]	12 (60%)
Ileum + Colon [*n* (%)]	8 (40%)
Length/cm [range; median]	1.6–21.3; 5
Smallest lumen circumference/cm [range; median]	0–7; 1.85
Bowel wall thickness/cm	11.28 ± 4.28
CDAI score	132–500; 243.5
History of bowel resection [*n* (%)]	6 (30%)
Treatments in the preoperative time [*n* (%)]	
Mesalamine	3 (15%)
Immunosuppressant	7 (35%)
anti-TNFα agents	8 (40%)

**Table 2:** Relative abundance of *Oscillospira* sp. in residual intestine segment and follow-up.

Patient ID	Relative Abundance of *Oscillospira* sp./‰	Groups	Follow-Up Time/Months	Outcomes
P1	0.209996	Low relative abundance	6	Recurrence
P2	0.380561	High relative abundance	11	Remission
P3	3.801072	High relative abundance	17.5	Remission
P4	0	Low relative abundance	17.5	Remission
P5	0.193879	Low relative abundance	12	Recurrence
P6	5.335989	High relative abundance	17.5	Remission
P7	0.058798	Low relative abundance	10	Recurrence
P8	1.568398	High relative abundance	15	Remission
P9	0.7292	High relative abundance	7.5	Remission
P10	0	Low relative abundance	12	Remission
P11	0	Low relative abundance	13.5	Remission
P12	46.098482	High relative abundance	10.5	Remission
P13	0	Low relative abundance	13.5	Remission
P14	0.549424	High relative abundance	15	Remission
P15	17.107977	High relative abundance	8	Remission
P16	1.055421	High relative abundance	12	Remission
P17	0	Low relative abundance	4.5	Remission
P18	0.764059	High relative abundance	5	Remission
P19	6.223602	High relative abundance	8	Remission
P20	0.125597	Low relative abundance	5	Remission

## Data Availability

The data that supports the findings of this study are available in Sequence Read Archive (SRA) of NCBI, the SRA accession number was SRR21830695, and the BioProject number was PRJNA887124.

## Supplementary Materials

The following supporting information can be downloaded at: https://www.mdpi.com/article/10.3390/microorganisms11030794/s1, Figure S1: Different kinds of bacterial relative abundances in all mucosal samples (Presented according to different taxonomy levels).; Figure S2: Random Forest analysis to identify distinguishing microbes at order (A) and class (B) level. The *x*-axis presented the contribution of each taxon to the microbial heterogeneity between stenotic and non-stenotic sites (shown by the mean decrease Gini index when corresponding taxon was removed from the analysis model). Statistical results of relative abundance variation between two sites for corresponding taxon: **● ***p* < 0.05; ● 0.05 < *p* < 0.1; ● *p* > 0.1. Figure S3: Comparison of genus *Oscillospira* abundance between CD patients and healthy controls in GMrepo database. Table S1: Results of Spearman Rank Correlation between bacterial abundance difference (abundance in stenotic site—abundance in non-stenotic site) and indicators.
