# Supplementary figures and images for: Mucosa-Associated Oscillospira sp. Is Related to Intestinal Stricture and Post-Operative Disease Course in Crohn’s Disease
Source: Microorganisms. 2023 Mar 20;11(3):794. doi: 10.3390/microorganisms11030794 (PMC10055919; doi:10.3390/microorganisms11030794)

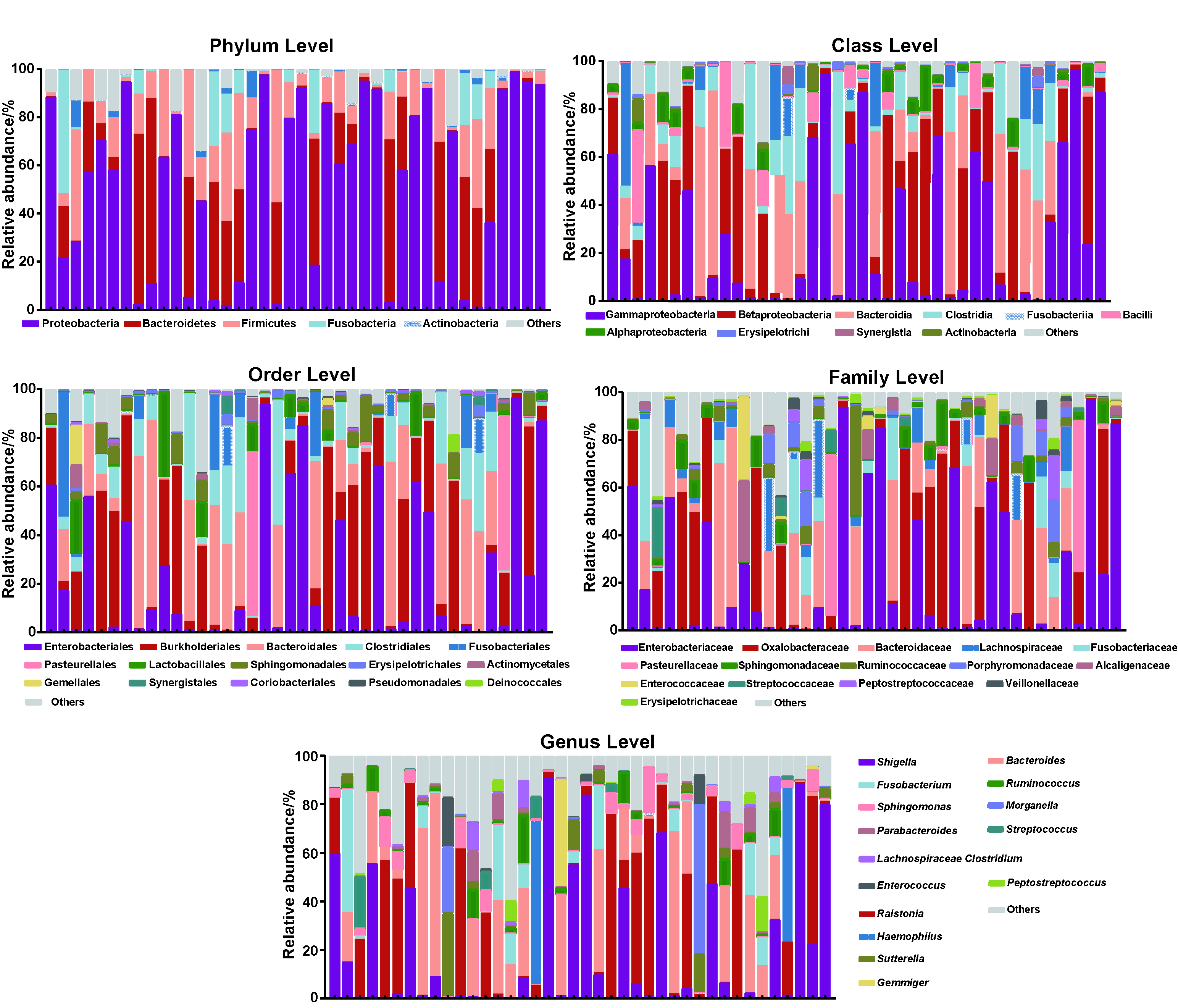

Supplement: Supplementary file 1 [file microorganisms-11-00794-s001.zip › Figure S1.tif]

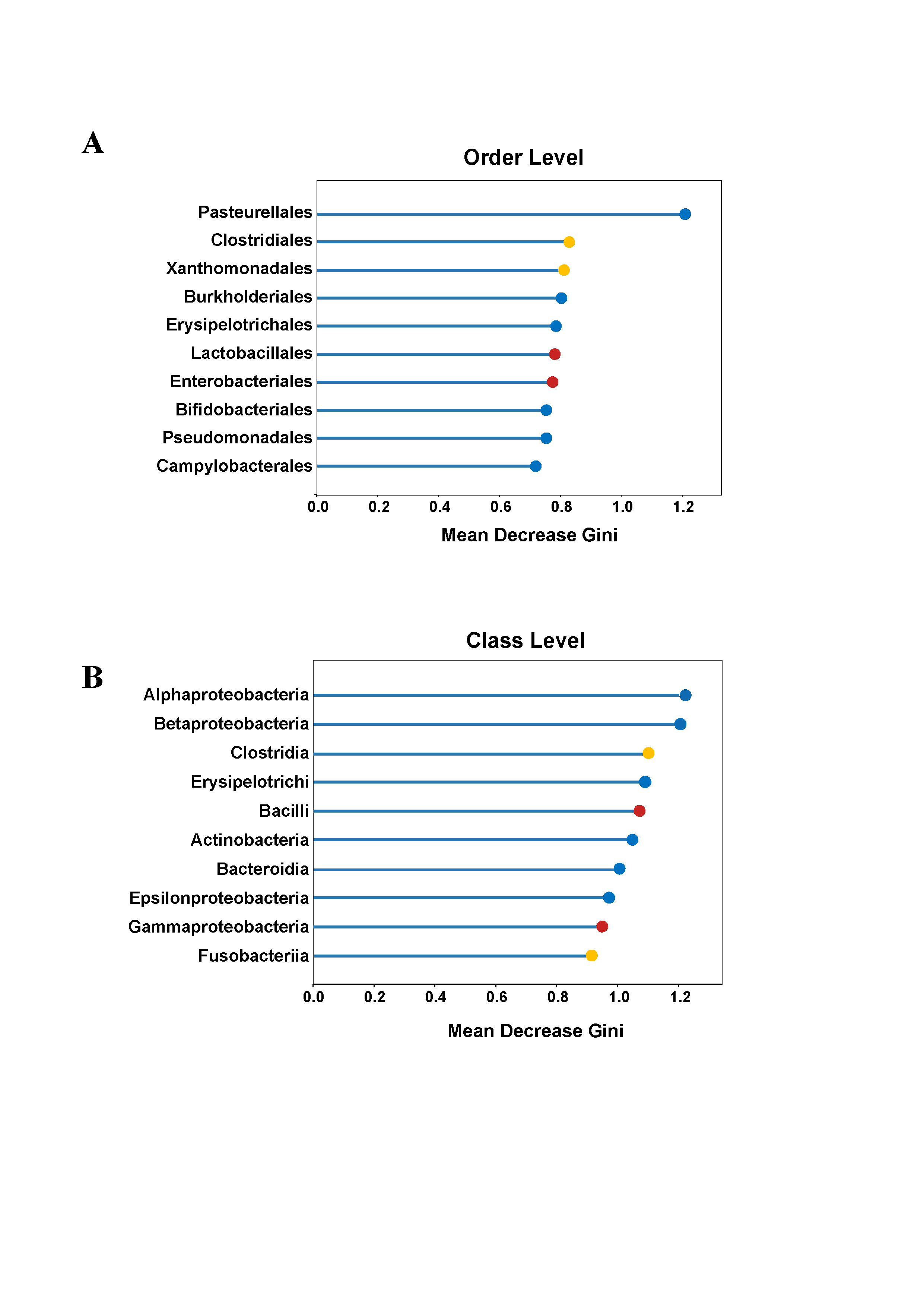

Supplement: Supplementary file 1 [file microorganisms-11-00794-s001.zip › Figure S2.tif]

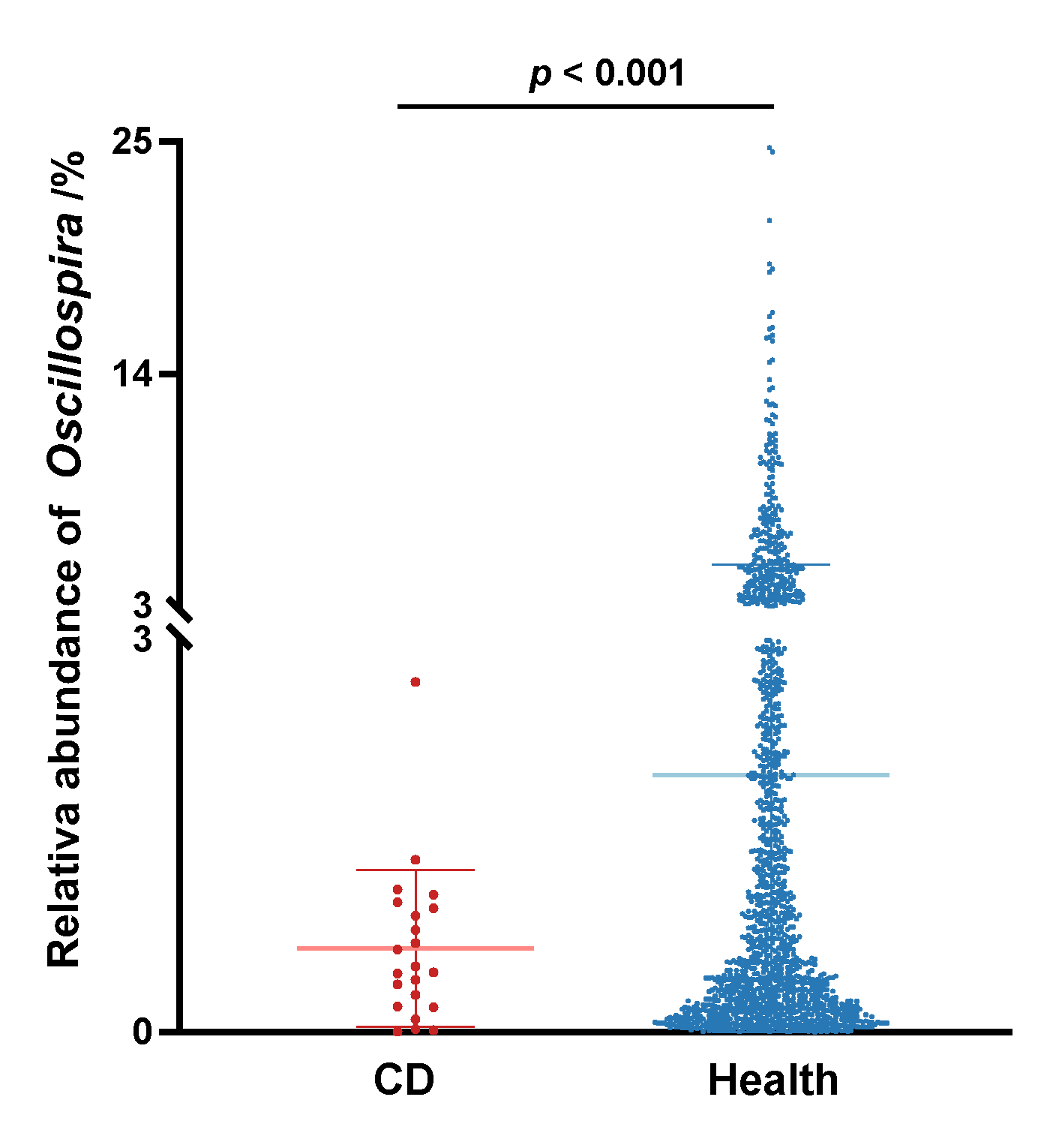

Supplement: Supplementary file 1 [file microorganisms-11-00794-s001.zip › Figure S3.tif]
